# Neural Processing and Production of Gesture in Children and Adolescents With Autism Spectrum Disorder

**DOI:** 10.3389/fpsyg.2019.03045

**Published:** 2020-01-22

**Authors:** Emily Fourie, Eleanor R. Palser, Jennifer J. Pokorny, Michael Neff, Susan M. Rivera

**Affiliations:** ^1^Department of Psychology, University of California, Davis, Davis, CA, United States; ^2^Center for Mind and Brain, University of California, Davis, Davis, CA, United States; ^3^Department of Neurology, University of California, San Francisco, San Francisco, CA, United States; ^4^Department of Computer Science, University of California, Davis, Davis, CA, United States; ^5^Department of Cinema and Digital Media, University of California, Davis, Davis, CA, United States; ^6^MIND Institute, University of California, Davis, Sacramento, CA, United States

**Keywords:** autism spectrum disorder, gesture, biological motion, fMRI, action

## Abstract

Individuals with autism spectrum disorder (ASD) demonstrate impairments in non-verbal communication, including gesturing and imitation deficits. Reduced sensitivity to biological motion (BM) in ASD may impair processing of dynamic social cues like gestures, which in turn may impede encoding and subsequent performance of these actions. Using both an fMRI task involving observation of action gestures and a charade style paradigm assessing gesture performance, this study examined the brain-behavior relationships between neural activity during gesture processing, gesturing abilities and social symptomology in a group of children and adolescents with and without ASD. Compared to typically developing (TD) controls, participants with ASD showed atypical sensitivity to movement in right posterior superior temporal sulcus (pSTS), a region implicated in action processing, and had poorer overall gesture performance with specific deficits in hand posture. The TD group showed associations between neural activity, gesture performance and social skills, that were weak or non-significant in the ASD group. These findings suggest that those with ASD demonstrate abnormalities in both processing and production of gestures and may reflect dysfunction in the mechanism underlying perception-action coupling resulting in atypical development of social and communicative skills.

## Introduction

Impairments in non-verbal communication are among the core features of autism spectrum disorders (ASD; American Psychiatric Association, 2013). In typical development, the use of deictic gestures, like pointing, emerges toward the end of the first year of life and representational gestures appear shortly thereafter (Capone and Mcgregor, 2004). In toddlers later diagnosed with ASD, retrospective and prospective research has demonstrated diminished spontaneous use of such gestures (Mitchell et al., 2006; Watson et al., 2013; Gordon and Watson, 2015). During the school-age years, gestures become increasingly more complex, scaffolding communication and language development (Capone and Mcgregor, 2004), Yet, children with ASD make more orientation and distortion errors (Dewey et al., 2007; Gordon and Watson, 2015) and show greater co-speech asynchrony while gestures (de Marchena and Eigsti, 2010). Compared to their typically-developing peers, individuals with ASD also show impairments in their ability to imitate others’ actions (Williams et al., 2004; Dewey et al., 2007), suggesting a deficit in both encoding and production. These gestural deficits appear to be only partially accounted for by basic motor impairments (Dewey et al., 2007; Dziuk et al., 2007; Zachor et al., 2010; Biscaldi et al., 2014).

Gestures are critically important to social interaction and interpersonal communication, and have been shown to have an influence on the development of pragmatic language skills (Miniscalco et al., 2014). Thus, deficits in this area can have a significant impact on daily functioning. Given the broad range of difficulties in gesture production among individuals with ASD, and their central role in social cognition, research is needed to characterize the nature of this impairment as well as to explore potential underlying mechanisms involved in this dysfunction.

One potential explanation for the impairments in non-verbal communication in ASD is a coincident deficit in visual sensitivity to biological motion (BM). BM is movement by an animate object; namely humans in the context of social cues. A growing body of evidence shows that individuals with ASD are compromised in their ability to detect BM (e.g., Atkinson, 2009; Koldewyn et al., 2010). This deficit appears to be one of degree, such that children with autism have significantly higher BM thresholds (Koldewyn et al., 2010). Additionally, those with ASD lack a typical preference for attending to BM stimuli, instead fixating more on scrambled or object motion (Annaz et al., 2012). These deficits are specific to BM perception, as perception of coherent motion (in the form of random moving dots) remains intact. Furthermore, performance on BM perception tasks correlates negatively with autism severity (Blake et al., 2003; van Boxtel et al., 2016; Blain et al., 2017). These findings suggest that abnormalities in attention to and processing of BM may be related to the development of atypical social cognition.

Movement provides complex information necessary for understanding and predicting reactions, emotions and intentions of others in social settings (Zilbovicius et al., 2006). As such, decreased visual sensitivity to BM may inhibit adequate processing and perception of dynamic non-verbal social cues, such as gestures. In turn, this dysfunction in the perceptual system may impede encoding, learning and subsequent performance of these actions, resulting in the observed gesture and imitation deficits. There is some evidence demonstrating a general link between perceptual and motor systems in ASD. Freitag et al. (2008) found that neuronal activity during perception of BM compared to scrambled motion was strongly associated with hand-finger imitation. Similarly, Price et al. (2012) showed that reduced visual sensitivity to human movement correlated with impaired motor skills in Asperger syndrome. These findings underscore the influence of BM perception abilities on the performance of complex motor tasks and suggest that action perception and action execution are closely linked.

The mechanisms underlying this perception-action coupling have been widely studied in typical and atypical development. The action observation network (AON) is comprised of two core regions, the inferior parietal lobule (IPL) and inferior frontal gyrus (IFG) as well as several other fronto-parietal cortical areas (Rizzolatti and Sinigaglia, 2010) and is active both during observation of an action and during execution of this action. This functional overlap allows the observer to encode the immediate goal of motor actions by mapping them onto one’s own behavioral repertoire and simulating a motor representation (Van Overwalle and Baetens, 2009). It is possible that dysfunction in this system may contribute to the disruption in the perceptual-motor coupling that hinders those with ASD from developing appropriate gestural and social communicative skills. Indeed, fMRI and EEG research in children and adolescents with ASD has revealed regions of the AON with abnormal patterns of neural activity during action observation/execution which is correlated with the severity of social impairment (Oberman et al., 2005; Dapretto et al., 2006; Iacoboni and Dapretto, 2006).

Another region known to be implicated in ASD and highly involved in social cognition is the posterior superior temporal sulcus (pSTS). This region acts as an orientation system, controlling attention to visual stimuli and routing visual input from both the motion-sensitive and object-characterizing visual areas to the AON (Puce and Perrett, 2003; Williams, 2008; Van Overwalle and Baetens, 2009). Additionally, in conjunction with the AON, it forms a core circuit for imitation (Iacoboni and Dapretto, 2006; Molenberghs et al., 2010). It supports processing of complex social information and is especially critical for processing of goal-directed actions such as gestures (Allison et al., 2000; Villarreal et al., 2008; Arfeller et al., 2013; Moessnang et al., 2017). Abnormalities in this region have been implicated in ASD: neuroimaging studies provide evidence of atypical patterns of activity during tasks involving social cognition, including those involving BM, as well as abnormal structural integrity (Zilbovicius et al., 2006; Pelphrey and Carter, 2008; Williams, 2008; Kaiser et al., 2010; Patriquin et al., 2016). Furthermore, many of these studies have demonstrated significant relationships between activity in pSTS and severity of social symptoms, such that less activity is associated with greater social deficits (Pelphrey and Carter, 2008; Kaiser et al., 2010; Alaerts et al., 2014).

Both the pSTS and AON are also involved in BM perception. Neural activity in response to BM compared to coherent motion has been localized to pSTS (Grossman et al., 2000; Puce and Perrett, 2003; Blake and Shiffrar, 2007). Compromised BM processing in ASD is supported through identification of its neural correlates. Investigations using fMRI have demonstrated that individuals with ASD show reduced activity in response to BM in pSTS as well as several parietal and frontal regions of the AON, while showing similar levels of activity during coherent or scrambled motion (Freitag et al., 2008; Koldewyn et al., 2011). EEG research has provided convergent evidence of disrupted neural mechanisms during BM processing (Kröger et al., 2014). Further, sensitivity to BM in these regions is linked to autism traits (Koldewyn et al., 2011).

A third region likely to be involved visuomotor representations of actions is the lateral occipital temporal cortex (LOTC), which contains regions that are sensitive to both body form (extrastriate body area, EBA) and body motion (middle temporal area, MT+). The EBA was originally identified as responding selectively to human body form (Downing et al., 2001) and is believed to play a role in understanding and inferring the goals and intentions from actions performed by a human agent (Peelen and Downing, 2007; Marsh et al., 2010). Its functional role has been expanded to encompass planning and execution of movement, indicating its involvement not only in perception but also in production of action (Astafiev et al., 2004; Ishizu et al., 2009; Oosterhof et al., 2010; Romaiguère et al., 2014; Zimmermann et al., 2016). This region overlaps significantly with region MT+, a primarily motion-sensitive area, important for BM processing, which has also shown to have body specific properties (Spiridon et al., 2006; Ross, 2014; Vangeneugden et al., 2014). The anatomical convergence of body, action, and motion selectivity suggests a likely function of the LOTC in the processing of dynamic and biologically relevant body and action representations. Some sparse research has indicated atypical activity in LOTC regions in young adults with ASD, particularly in the left EBA (Okamoto et al., 2014, 2017, 2018), but further research is needed to investigate this dysfunction.

Given the critical role that LOTC, pSTS and the AON play in social perception, BM and body form processing as well as their documented dysfunction in ASD, these regions make good candidates for studying a potential disruption of perception-action coupling in ASD. Dysfunction in neural mechanisms linking observation and execution of action could hinder transformations from perception to action and result in a cascade of detrimental effects including impaired imitative and gestural skills as well as social-communicative deficits. This study investigates this putative link between processing and production of familiar actions to determine whether faulty BM perception may underlie the broader social impairment in ASD.

### The Current Study

Much of the existing BM research uses point-light displays, consisting of coordinated moving dots that represent joints of a human performing an action. These are coarse, simplistic low-level stimuli that are likely processed early in the visual stream. This study extends the literature demonstrating BM deficits in ASD by using complex stimuli that more closely mimics real-world social interaction. Videos of human-like avatars were rendered from digitalized motion capture of actions performed by human models and presented to participants during an fMRI task. Given previous empirical findings, we predict that, compared to typically developing (TD) participants, participants with ASD will show hypoactivation of areas involved in BM and gesture processing, namely LOTC, pSTS, and the fronto-parietal AON.

The current study also aimed to investigate the sensitivity of BM perception in ASD. As noted above, the BM deficit in ASD appears to be one of degree, as individuals with ASD have higher thresholds for detecting BM in noise compared to their TD counterparts. Therefore, stimuli were parametrically manipulated in the amount of movement contained in the action, to assess sensitivity of both behavioral and neural responses to different levels of movement intensity. We hypothesized that those with ASD would require greater movement intensity to recognize gestures and to elicit levels of neural activity, comparable to TD controls.

In addition to assessing neural correlates of gesture processing, quality of gesture production was evaluated in a charade-style paradigm outside of the scanner. This component allowed us to examine differences in the way that children and adolescents with ASD and controls produce familiar actions. We hypothesized that individuals with ASD would make more errors while performing gestures. Lastly, the study examined the brain-behavior relationships between gesture processing, gesture production and various behavioral symptoms of ASD. We hypothesized that quality of gesture performance would be positively correlated with neural activity during gesture observation, and that both of these measures would be negatively associated with autism symptomology.

In summary, this study is a multi-disciplinary examination of the brain and behavioral underpinnings of gesture processing among children and adolescents with ASD. The complementary components shed light on individuals’ sensitivity to gestural displays, visual processing and performance of familiar gestures, and brain-behavior relationships between these measures in order to elucidate the connection between abnormal perceptual processes and the affected outcomes in individuals with ASD.

## Materials And Methods

### Participants

A total of 19 children and adolescents (4 females) with a diagnosis of autism and 20 age and IQ matched typically developing (TD) children and adolescents (3 females) participated in the study. Autism diagnosis was confirmed by meeting both DSM-IV criteria and the cutoff score on the Autism Diagnostic Observation Schedule (ADOS; Lord et al., 2000). An additional 9 participants (7 ASD, 2 TD) were recruited for this study but excluded on the basis of study criteria (IQ below 80, co-occurring neurological or medical conditions, preterm birth, use of anti-psychotic medications, MRI contraindications including braces, glasses and other implants or devices. Performance IQ, assessed with the Weschler Abbreviated Scale of Intelligence (WASI; Wechsler, 1999), was not significantly different between groups, however, verbal IQ was marginally higher in the TD group. All participants had normal or corrected to normal vision. Participant demographics are displayed in Table 1.

**TABLE 1:** Demographics of ASD and TD groups for full sample (*N* = 39).

	**ASD**		**TD**		
	**Mean (*SD*)**	**Range**	**Mean (*SD*)**	**Range**	***p***
Age	13.47 (1.68)	10.5–15.8	12.73 (2.32)	9.6–16.9	0.27
PIQ	107.06 (14.10)	85–131	110.26 (12.50)	80–125	0.60
VIQ	106.84 (13.20)	82–127	115.11 (16.04)	88–137	0.09
ADOS	8.93 (1.98)	7–13	NA		
SRS	76.68 (15.33)	48–90	44.16 (7.75)	35–62	< 0.0001
SCQ	22.00 (7.08)	11–32	2.21 (2.84)	0–9	< 0.0001
DCDQ	41.63 (12.41)	23–72	70.47 (7.06)	51–75	< 0.0001

**Participants age in years, IQ scores on performance (PIQ) and verbal (VIQ) domains, scores on the Autism Diagnostic Observation Schedule (ADOS), Social Responsiveness Scale (SRS), Social Communication Questionnaire, Developmental Coordination Disorder Questionnaire (DCDQ).**

The fMRI analyses included 15 individuals with ASD (3 left-handed) and 16 TD participants (1 left-handed). Data from eight additional participants was not included in the final analyses because they were unable to finish the scanning protocol (ASD *N* = 1, TD *N* = 2), had excessive movement (>3.4 mm) in the scanner (ASD *N* = 2, TD *N* = 1) or were not reliably engaged during the task (ASD *N* = 1, TD *N* = 1). The behavioral video portion of the study was completed by 12 participants with ASD and 16 TD individuals. A subset of 8 participants with ASD and 12 TD participants provided data for both the fMRI and video portions of the study and were used for an exploratory assessment of brain-behavior relationships between production and processing of gestures. Subsamples for each of these analyses were matched on age and performance IQ.

Participants were recruited through the subject tracking system at the University of California Davis MIND Institute. Individuals were screened and excluded for co-occurring neurological conditions (seizures, Tourette’s syndrome), medical disorders associated with autism (fragile X Syndrome), preterm birth and use of anti-psychotic medications. TD participants had no history of developmental delay or immediate family member diagnosed with ASD. Guardians of participants signed an informed consent approved by the University of California Davis Institutional Review Board prior to inclusion in the study.

### Behavioral Measures

Several parent-report measures were administered to assess social and motor functioning. The Social Communication Questionnaire, Lifetime version (SCQ; Rutter et al., 2003) is a 40-item questionnaire, with higher scores indicating greater social difficulties. Scores greater than or equal to 15 denote possible ASD. The Social Responsiveness Scale (SRS; Constantino, 2005) is used to identify presence and severity of social impairment, specifically within ASD, with 65 items on a 4-point Likert scale. Higher scores indicate greater impairment; scores 60 and above indicate some degree of social deficiency. The Developmental Coordination Disorder Questionnaire (DCDQ; Wilson et al., 2009) is designed to screen for motor coordination disorders. The questionnaire includes 15 items on a 7-point Likert scale with lower scores indicating greater impairment in motor coordination.

### fMRI Task

**FIGURE 1:**
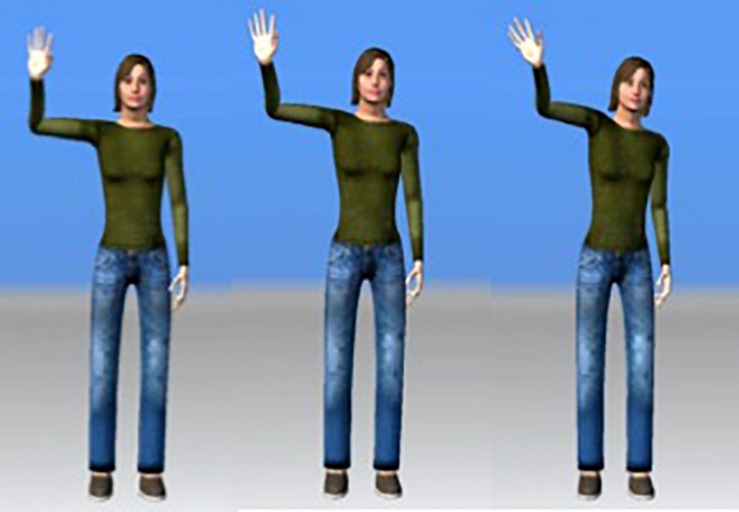
Example of “wave” stimuli at three levels of movement (still frames captured at the height of the action). Subtle displayed on the left and exaggerated on the right.

Stimuli consisted of 5 s long videos depicting animations of 11 distinct action gestures. These gestures were originally performed by a human actor, recorded with optical, marker-based motion capture and rendered to a 3D human avatar. Two different types of gestures were presented: functional pantomimes (*N* = 5; e.g., driving, lifting) and communicative gestures (*N* = 6; e.g., waving, scolding) to determine whether gestures that were social in nature, i.e., communicative, elicited differential responses in our ASD group compared to TD controls. The gestures were computationally manipulated to vary in the scale of the movement, yielding three levels, from subtle to exaggerated (Figure 1). Each gesture was presented at each intensity level twice over the course of two functional runs, each run lasting approximately 7 min. Following presentation of each gesture, two words corresponding to actions in the videos appeared on the screen, one on the left and one on the right. Participants were tasked with choosing one of the two options that most closely matched the gesture presented and made their selection on a button box.

### Image Acquisition and Preprocessing

MR data were acquired on a 3.0T Siemens Trio scanner with a Siemens 8-channel head coil at the UC Davis Imaging Research Center. Functional MR was acquired using a standard echo planar pulse sequence with the following parameters: TR = 2000 ms, TE = 25 ms, FA = 90°, FOV = 305, matrix, 36 axial slices, voxel size = 3.4 mm^3^. During the same scan session, structural images were obtained using a T1-weighted MPRAGE 3D MRI sequence (TR = 2170 ms, TE = 4.86 ms, flip angle = 7°, FOV = 256 mm × 256 mm, 1 mm slice thickness). Presentation software (Neurobehavioral Systems, Inc., Berkeley, CA, United States) was used to present the functional task, which was projected onto a screen located at the participant’s feet and viewed through a head-mounted mirror.

Data preprocessing and analysis was performed with statistical parametric mapping software, SPM12^1^ run within MATLAB (r2014b; Matlab Mathwork, Inc., Natick, MA, United States). Preprocessing of images was completed using standard procedures including slice-time correction, realignment, coregistration, normalization to stereotaxic Montreal Neurological Institute (avg152 T1-weighted template) and smoothing with a 5 mm Gaussian kernel to decrease spatial noise. Motion parameters for each subject, obtained during the realignment step, were added into the model as additional regressors; these did not differ across groups.

### Gesture Production Task

The behavioral task resembled a game of charades. Participants were instructed to pantomime an action by means of a written instruction handed to them on a small card. The gestures included three communicative gestures (e.g., Hurry up, Stop, and Shush/Be quiet) and 12 functional gestures (e.g., Brush teeth, Comb hair, Use scissors to cut paper, Dribble a basketball, Erase a chalkboard, Hammer a nail, Jump rope, Painting a wall, Saw a piece of wood, Scoop ice cream, Unlocking a door with a key, and Write on a piece of paper). Participants were allowed to perform the action by whatever means they deemed suitable. A confederate in the room was required to guess the action performed. This was designed to encourage the children’s engagement in the task and allowed them to attempt the action multiple times if the first try was not guessed successfully. Each gesture represented a trial, thus participants completed 15 trials. These sessions were video-recoded for later analysis.

### Data Analysis

#### fMRI

Single subject effects were estimated using the general linear model in SPM12. Each trial was modeled with the canonical hemodynamic response function over the duration of the video. Regressors were included to account for participant head movement. Second level analyses consisted of one and two-sample *t*-tests for each contrast of interest. Significant clusters of activation were determined using a primary cluster-forming threshold of *p* < 0.001, with an extent of 20 voxels. Only clusters surviving FWE-correction at a threshold of *p* < 0.05 were reported as significant.

Additionally, several region of interest (ROI) analyses were performed to examine levels of activity in specific areas. ROIs for the LOTC were functionally-defined for each individual and were created by drawing 6 mm radius spheres around the most significantly activated “peak” voxel within a restricted part of the cortex based on anatomical locations previously reported in research involving human bodies (Right LOTC: 46, −70, −1; L LOTC: −52, −72, 4; Downing et al., 2006, 2007; Okamoto et al., 2014, 2017). Two masks were also created to capture activity in bilateral pSTS and the AON network. An atlas-based definition of pSTS based on McNorgan et al. (2013) was used. It was outlined as the intersection of the AAL superior temporal and middle temporal gyri, each dilated by 4 mm along each axis, defining the sulcus. Subject wise local maxima were extracted from the posterior third of this region, greater than *y* = −40 and 6 mm radius spheres were drawn. Lastly, an AON mask was created according to activation areas reported to be correlated and consistently engaged during observation of hand and arm actions (Shaw et al., 2012) and used in previous studies with TD and ASD adolescents (Pokorny et al., 2015, 2018). The mask included 6 mm radius spheres around coordinates of 10 bilateral areas inferior frontal gyrus (IFG: −50,12,22; 50,16,24), premotor cortex (PMC: −40,−2,45; 42,2,44), inferior parietal lobule (IPL: −42,−41,47; 37,−42,49), supramarginal gyrus/angular gyrus (SMG/AG: −58,−28,34; 50,−30,42), and superior parietal lobule (SPL: −28,−56,56; 26,−56,60). For each of the three ROIs, mean parameter estimates (beta) were extracted by averaging the parameter estimates of all voxels that fell within the defined region using MarsBaR^2^. This process was performed for each of the six main conditions (2 gestures types at 3 movement levels), compared to baseline, and entered into a 3-way, group by gesture type by movement intensity ANOVA.

#### Behavioral Coding

A coding scheme was developed in-house. All trials were double scored by two of the authors (ERP and EF), who were trained and calibrated on the coding scheme. The scorers were blind to the participants’ diagnosis and their only experience with the participants was through watching the videos. All gestures were scored for the following criteria: (1) events acted out, (2) gaze, (3) body positioning, (4) limb movement, (5) hand posture, (6) use of space, (7) tempo, (8) the ability of the scorer to determine the meaning of the gesture, and (9) the overall quality of the gesture, on a three-point scale of 0–2, where 2 represented accurate performance of the gesture and 0 represented major errors in the portrayal of the gesture (see Supplementary Appendix for full coding scheme). On videos where raters disagreed on total score by more than 3 (19% of all videos), scores were discussed until consensus was reached. All other scores were averaged between raters. All gestures were also coded as “yes” or “no” for the presence of (10) use of own body as an object (e.g., using their own hand to symbolize a piece of paper), (11) miming the use of an imaginary object (e.g., using a hand posture appropriate for holding a toothbrush), (12) use of environmental context (e.g., using furniture or the wall), (13) embellishing the gesture with additional context (e.g., licks the ice cream after scooping it) and (14) whether there were multiple attempts to perform the gesture.

#### Behavioral Analysis

All data were screened for normality using Kolmogorov–Smirnov tests. Appropriate non-parametric tests (Mann–Whitney *U*-tests) were applied in any instances in which the assumption of normality was violated and for items 10–14 where responses were ordinal; otherwise, independent *t*-tests were used. Cohen’s d is given as an indication of effect size. One ASD participant was missing gaze codes for all actions performed, and another was missing all except two gaze codes. These two participants are thus not included in the analysis of gaze. They are included in the analysis of participants’ overall total score, although the analysis is also reported with these two participants excluded. Participants’ overall gesture performance (total score) was calculated by summing performance across all 15 gestures on the codes listed (items 1–9 above): events acted out, gaze, body positioning, limb movement, hand posture, use of space, tempo and meaning. Higher scores indicate better performance. This analysis was also repeated with meaning excluded. Items 10 through 14 were not included when calculating overall performance, as their presence does not necessarily indicate better performance because participants were not given instructions on how to execute the action.

#### Correlation Analyses

Correlations between fMRI and behavioral video data were examined by calculating Pearson correlation coefficients between beta values extracted from ROIs and overall gesture performance scores. These analyses were run separately for each group. Associations between neural activity during the task, gesture performance and behavioral measures was also assessed. Additionally, given the large age range of participants in the study, we examined the relationship between age and both neural activity and gesture performance to account for developmental changes.

## Results

### Behavioral Measures

Scores on both the SCQ, which assesses social functioning, and the SRS, which indexes social responsiveness, were significantly higher in the ASD group compared to the TD group, indicating greater social difficulties (Table 1). Scores on the DCDQ’07, which assesses motor functioning, were significantly lower in the ASD group compared to the TD group, indicating poorer parent-reported motor skills in the ASD group (Table 1).

### Gesture Performance

Significantly higher overall performance was seen in the TD group (*M* = 204.62) than the ASD group (*M* = 185.42) [*t*(14.98) = 2.36, *p* = 0.03, *d* = 0.99]. The same pattern of results was also seen when excluding the meaning code, with higher overall performance in the TD group (*M* = 183.95) than the ASD group (*M* = 167.97) [*t*(14.05) = 2.50, *p* = 0.03, *d* = 1.08]. Because the meaning code was not an entirely unbiased rating (i.e., raters were aware of which action they were viewing) and similar results were shown for analyses including and excluding this code, we decided to use the overall performance scores without meaning for all subsequent analyses.

To explore whether the significant difference in overall gesture performance between ASD and TD participants was being driven by performance on certain aspects of the gestures, we tested for significant group differences on all codes. Table 2 shows the average values for each group as well as significance values and effects sizes for the group comparisons. Significant group differences emerged for quality of the gestures and hand posture codes, with the TD group demonstrating significantly better performance than the ASD group. Additionally, there was a significant group difference in the use of environmental context; participants with ASD more frequently used environmental context (such as furniture or the wall) to perform their gestures than their TD counterparts. No other codes showed significant group differences.

**TABLE 2:** Behavioral performance of gestures by group.

**Code**	**ASD**	**TD**	***p***	***d***
Events acted out	26.69 (3.12)	27.70 (1.93)	0.60	0.40
Gaze	24.57 (4.33)	26.58 (3.55)	0.21	0.51
Body positioning	27.75 (1.86)	28.65 (2.41)	0.60	0.42
Limb movement	22.15 (3.73)	23.56 (2.15)	0.60	0.48
Hand posture	23.21 (2.21)	26.01 (2.21)	0.04*	0.89
Use of space	26.05 (3.00)	28.12 (1.54)	0.13	0.91
Tempo	22.46 (3.28)	22.97 (2.98)	0.67	0.17
Quality	15.99 (5.88)	20.69 (3.35)	0.01*	1.02
Uses own body as object	1.69 (1.01)	1.70 (1.39)	0.98	0.01
Mimes imaginary object	11.37 (0.77)	11.15 (0.74)	0.45	0.29
Embellishes with added context	3.43 (1.85)	3.01 (2.09)	0.58	0.62
Uses environmental context	2.86 (1.74)	1.46 (1.97)	0.04*	0.75
Makes multiple attempts	0.76 (0.76)	0.74 (0.70)	0.91	0.02

Average values for each code, as well as significance values and effects sizes for group comparisons. *p < 0.05.

#### Gesture Performance and Age

In the TD group, gesture performance was positively associated with age (*r* = 0.61, *p* = 0.01), but this association was not found in the ASD group (*r* = 0.24, *p* = 0.46). In order to determine whether differential age effects were accounting for observed group difference in gesture performance, we performed a one-way ANCOVA with age as a covariate. There were, however, no main or interaction effects with age suggesting that gesture performance was dependent solely on group status [effect of group: *F*(1, 23) = 8.94, *p* = 0.01]. While typical children seem to improve in gesture performance with age, the ASD group does not show the same age-related progress. However, this differential age effect was not strong enough to indicate a significantly different developmental trend in the two groups.

### fMRI Task

#### Behavioral Data

To determine whether individuals with ASD differ from TD individuals in their ability to recognize gestures, we used a three-way ANOVA with two within-subject factors: level of movement intensity (subtle, mid-level, exaggerated) and gesture type (functional, communicative) and a between-subjects factor of group status (ASD, TD). Four participants (all TD) were excluded from this analysis as their responding was at chance, due to difficulty understanding the task instructions or the use of incorrect response keys, leaving 12 TD and 15 ASD participants for analysis.

For reaction time, there were no significant main effects of group or interactions with group, suggesting that the ASD and TD groups did not systematically differ in the speed at which they recognized gestures, for level of movement or gesture type. There was, however, a main effect of gesture type, *F*(1, 25) = 17.92, *p* < 0.001, a main effect of movement intensity, *F*(2, 50) = 21.93, *p* < 0.00001, and an interaction between type and movement, *F*(2, 50) = 5.64, *p* < 0.01. Follow-up comparisons for the main effect of gesture type revealed that communicative gestures (*M* = 1.20) took longer than functional gestures (*M* = 1.10) to identify, *p* < 0.0001. Follow-up pairwise *t*-tests for the effect of movement intensity demonstrated that across both gesture types, the subtle gestures (*M* = 1.25) took longer to recognize compared to both mid-level (*M* = 1.09), *p* < 0.0001, and exaggerated gestures (*M* = 1.12), *p* < 0.0001. Follow-up comparisons for the interaction revealed that communicative gestures elicited longer reaction times than functional gestures only for mid-level (*p* < 0.0001) and exaggerated gestures (*p* = 0.05).

As with reaction time, accuracy scores showed no main effect nor interactions involving group, suggesting similar performance across groups on the recognition task. There was a main effect of gesture type, *F*(1, 25) = 31.34, *p* < 0.00001, and a main effect of movement intensity, *F*(2, 50) = 4.64, *p* = 0.04. Pairwise comparisons between the two gesture types revealed that functional gestures (*M* = 0.95) were more accurately recognized than communicative gestures (*M* = 0.91), *p* < 0.0001. Follow-up comparisons between each level of movement intensity showed that participants recognized mid-level gestures (*M* = 0.95) more accurately than exaggerated (*M* = 0.92), *p* < 0.01, and marginally better compared to subtle gestures (*M* = 0.92), *p* = 0.05.

#### Whole Brain Data

##### Neural response to all gestures

One-sample within-group *t*-tests revealed large clusters of activation (*k* = 150+) in bilateral LOTC in both groups (MNI coordinates: L LOTC: *x* = −49, −71, 5; R LOTC: *x* = 44, *y* = −78, *y* = −2). The TD group showed additional activations in several smaller clusters (*k* = 27+) located in the left hemisphere including the premotor cortex, supplementary motor area, insula and thalamus. The ASD group showed one additional cluster of activity (*k* = 80) in the left inferior frontal gyrus. However, a two-sample between group *t*-test revealed no regions in which there was significantly greater activation in either group (TD > ASD, ASD > TD) at a cluster level threshold of *p* = 0.05 (FWE corrected). See Table 3 for areas of significant activation.

**TABLE 3:** Brain areas of significant activation for each group in response to all gestures, the effect of gesture type and movement intensity across both groups.

					**MNI coordinates**		
**Contrast and group**	**Region**	***t*-value**	**Z-score**	***k***	***x***	***y***	***z***
**All gestures > baseline**							
TD	R lateral occipital temporal cortex	9.88	5.53	353	44	−78	−2
	L lateral occipital temporal cortex	8.01	5.01	347	−53	−64	5
	L premotor cortex	7.35	4.79	82	−35	5	32
	L thalamus	7.75	4.93	32	−18	−33	−2
	L insula	6.42	4.45	30	−32	22	−2
	L supplementary motor area	5.85	4.22	27	−4	12	49
ASD	L lateral occipital temporal cortex	9.05	5.22	159	−35	−91	−5
	R lateral occipital temporal cortex	7.97	4.91	266	44	−84	5
	L inferior frontal gyrus	5.58	4.04	80	−42	19	25
**Effect of gesture type**							
CG > FA	L inferior frontal gyrus	6.47	5.14	141	−46	29	−5
	L middle temporal gyrus	6.21	4.99	94	−42	1	−22
	L cingulate gyrus	5.82	4.77	147	−8	−50	29
	L middle temporal gyrus	5.81	4.76	101	−53	−36	1
	L supplementary motor area	5.29	4.45	51	−4	12	59
	R middle temporal gryus	4.65	4.03	48	47	−40	1
FA > CG	–						
**Effect of movement intensity**							
Subtle > exaggerated	L caudate	4.96	4.21	73	−11	1	18
Exaggerated > subtle		–					

**Only areas of significance are reported and the following information provided: T-value, Z-score, cluster size and coordinate of peak activation in MNI coordinates. (CG, communicative gesture; FA, functional gesture).**

##### Effect of gesture type

No regions showed significantly greater activity for the functional compared to communicative gestures. When looking at the communicative > functional contrast (cluster level FWE correction of *p* < 0.05) several regions appeared showing stronger activation. These regions included several clusters in the left hemisphere including the inferior frontal gyrus, supplementary motor area, posterior cingulate, and middle temporal area (BA 21). Between group comparisons (TD > ASD, ASD > TD) revealed no significant differences of the effect of gesture type across the whole brain.

##### Effect of movement intensity

We performed *t*-tests both within and between groups using a contrast to detect parametric differences in activity based on intensity of movement (−1/1 = subtle, 0 = mid-level, 1/−1 = exaggerated). At a cluster level FWE corrected threshold of *p* < 0.05, one cluster, the left caudate nucleus, showed greater activity for the subtle compared to exaggerated gestures. There were no areas of significant difference in activation between groups.

#### ROI Analyses

ROI analyses were performed to investigate whether neural activity was sensitive to gesture type or movement level across groups within the following *a priori* regions: LOTC, pSTS, and AON.

##### LOTC

**FIGURE 2:**
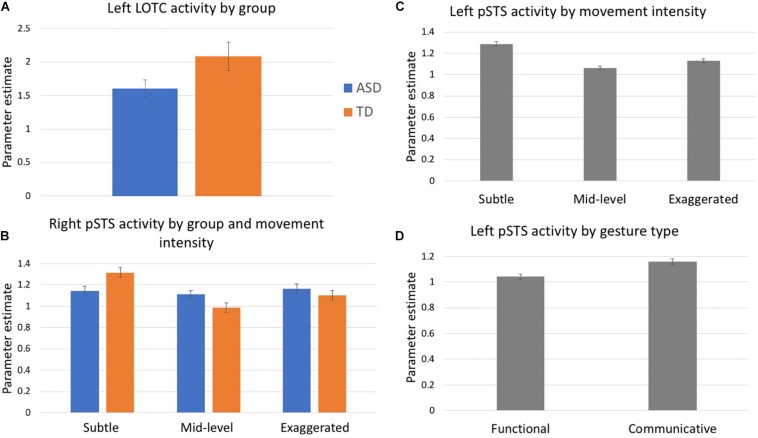
Mean parameter estimates extracted from ROIs: **(A)** Left LOTC, **(B)** Right pSTS, and **(C,D)** Left pSTS. Blue denotes participants with ASD, orange indicates controls, gray indicates values collapsed across both groups.

In a 3-way ANOVA (movement × type × group), left LOTC showed a trending main effect of group, *F*(1, 29) = 3.43, *p* = 0.08 (Figure 2A). There were no additional main effects of either gesture type or movement level nor an interaction effect with group. No main or interaction effects were detected in the right LOTC.

##### pSTS

Analyses of activity in the right pSTS ROI revealed a group by movement intensity interaction, *F*(2, 58) = 3.13, *p* = 0.05 (Figure 2B). Follow-up comparisons show that the TD group had differential activation based on the level of movement intensity. The subtle gestures elicited greater activity than the mid-level (*p* = 0.01) and marginally greater activity than exaggerated gestures (*p* = 0.05). However, these differences did not exist in the ASD group. The group by type interaction was marginally significant, *F*(1, 29) = 3.27, *p* = 0.08. There were no other significant main effects or interactions.

Left pSTS showed a main effect of movement intensity, *F*(2, 58) = 3.44, *p* = 0.04 and follow-up comparisons showed that subtle gestures elicited greater activity than mid-level gestures, *p* = 0.03 (Figure 2C). This trend was driven by data in the TD group, for whom subtle gestures elicited greater activity than both mid-level (*p* = 0.02) and exaggerated (*p* = 0.01) gestures, while no differential activity based on movement intensity was present in the ASD group, however, the group x movement interaction was not significant, *p* = 0.30. There was also a main effect of gesture type, *F*(1, 29) = 8.76, *p* = 0.001, with all participants exhibiting greater activity for communicative than functional gestures (Figure 2D). No other main effects or interactions were detected.

##### AON

AON analyses demonstrated a marginal trend for the effect of movement intensity, *F*(2, 58) = 2.42, *p* = 0.09. There were no other main or interaction effects with group.

##### Neural activity and age

We examined the relationship between age and activity in each of the ROIs. In the TD group, there were significant positive associations between age and activity in two regions: left pSTS (*r* = 0.51, *p* = 0.04) and AON (*r* = 0.64, *p* = 0.01). No significant associations emerged between neural activity and age in the ASD group.

### Relationships Between Neural Activity, Gesture Performance, and Behavioral Measures

#### Neural Activity and Gesture Performance

**FIGURE 3:**
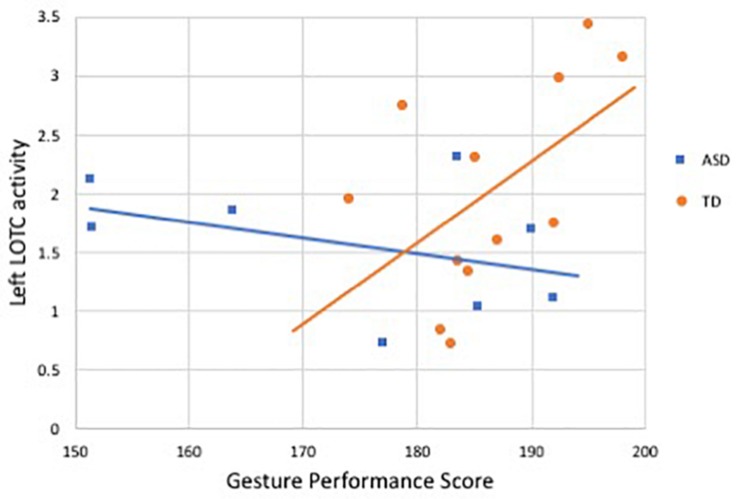
Correlation between activity in left LOTC during gesture processing and gesture performance score in ASD and TD groups. Blue denotes participants with ASD (*r* = −0.40, *p* = 0.31), orange indicates controls (*r* = 0.59, *p* = 0.04).

In the subset of participants who completed both the fMRI and behavioral components (8 ASD, 12 TD), we performed exploratory analyses examining correlations between neural activity and gesture performance. Activity in left LOTC was positively correlated with the total gesture score in the TD group (*r* = 0.59, *p* = 0.04), but no significant relation was found in right LOTC (*r* = 0.31, *p* = 0.32), nor with either region in the ASD group (L LOTC: *r* = −0.40, *p* = 0.31; R LOTC: *r* = 0.20, *p* = 0.63); see Figure 3. Fisher’s transformation showed that the difference between these coefficients was significantly different between groups (*p* = 0.04). Correlations with activity in the AON showed a similar trend: in the TD group, gesture performance was positively related to activity in the AON mask region (*r* = 0.57, *p* = 0.05), while no relation was present in the ASD group (*r* = −0.11, *p* = 0.80). Given the association between age and AON activity, we then included age as a covariate using partial correlation and this relationship in the TD group was no longer significant (*r* = 0.07, *p* = 0.83). No significant correlations were detected between activity in bilateral pSTS or AON and gesture performance in either group.

#### Neural Activity and Behavioral Measures

##### Social impairment

In the TD group, activity in the left LOTC was negatively correlated with SRS scores (*r* = −0.49, *p* = 0.05). There was also a negative but non-significant association between left LOTC activity and SCQ scores in the TD group (*r* = −0.28, *p* = 0.19). The ASD group showed no significant relationships between left LOTC activity and SRS (*r* = −0.09, *p* = 0.76) or SCQ scores (*r* = −0.19, *p* = 0.49). Similarly, activity in the AON was negatively correlated with both measures of social functioning only in the TD group (SRS: *r* = −0.47, *p* = 0.05; SCQ: *r* = −0.50, *p* = 0.05) but not the ASD group (SRS: *r* = −0.16, *p* = 0.57; SCQ: *r* = 0.05, *p* = 0.87). However, Fisher’s transformation revealed that none of the correlation coefficients were significantly different between groups. There were no significant associations between activity in right LOTC or bilateral pSTS and any of the measures of social impairment in either group.

##### Motor coordination

There were no significant relationships between DCDQ scores and neural activity in any region in either group.

#### Gesture Performance and Behavioral Measures

##### Social impairment

**FIGURE 4:**
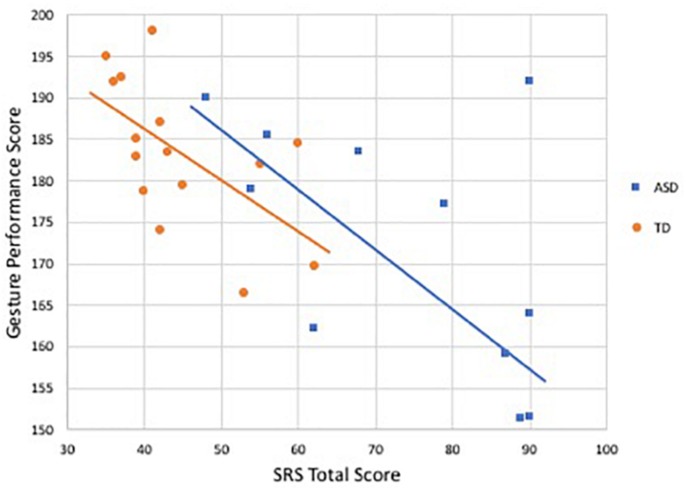
Relationship between SRS total score and gesture performance score by group. Blue denotes participants with ASD (*r* = −0.55, *p* = 0.07), orange indicates controls (*r* = −0.60, *p* < 0.05).

Gesture performance was negatively correlated with SRS scores in both groups, reaching statistical significance only in the TD group (*r* = −0.60, *p* = 0.02) and trending toward significance in the ASD group (*r* = −0.55, *p* = 0.07); see Figure 4. Similar results were observed for the relationship with SCQ (TD: *r* = −0.54, *p* = 0.03; ASD: *r* = −0.54, *p* = 0.08).

##### Motor coordination

In the TD group, gesture performance was correlated with motor coordination, such that greater motor coordination was associated with better performance of gestures (*r* = 0.65, *p* = 0.01). No such associations were demonstrated in the ASD group (*r* = 0.34, *p* = 0.30); yet, this coefficient was not statistically different from that of the TD group.

## Discussion

The present study set out to investigate differences in behavioral and neural sensitivity to action gestures and gesture production abilities in a group of children and adolescents with ASD compared to age- and performance IQ-matched TD counterparts. Further, associations between these measures and social difficulties were assessed.

### Neural Activity During Gesture Processing

The primary aim of this study was to examine the underlying neural correlates of gesture processing and determine whether our groups showed differential neural sensitivity to movement intensity or gesture type. We predicted that participants with ASD would show hypoactivation of areas involved in BM and gesture processing, however there were no significant main effects of group in any ROIs. Left LOTC showed a marginal main effect of group, where previous research has demonstrated attenuation of activity in LOTC and EBA in ASD (Okamoto et al., 2014, 2018). LOTC showed no effects of movement intensity and gesture type, suggesting that it may be performing more basic, early-stream processing, without differential sensitivity to the amount of movement or the type of gesture being observed. Studies examining networks involved in visual processing of body parts suggest that this region plays a role in integrating form and movement information about bodies and actions and relaying this to associative parietal and temporal regions in order to perform action (Astafiev et al., 2004; Zimmermann et al., 2018), which may explain the lack of differential activity by movement and gesture type.

On the other hand, pSTS did demonstrate modulation of activity by both movement intensity and gesture type. Right pSTS showed differential activity based on movement intensity only in the TD group, with greater activity elicited for subtle gestures, and a trending group by gesture type interaction. Groups demonstrated similar patterns of sensitivity to both movement intensity and gesture type in the left pSTS, with comparatively greater activity elicited by both subtle and communicative gestures. Whole brain analyses showed consistent findings, with heightened activity in a few frontal regions, namely IFG and SMA, during presentation of communicative compared to functional gestures, and in one area, the caudate nucleus, for subtle compared to exaggerated gestures, suggesting that these two factors necessitated higher processing demands.

Given the role of the pSTS in controlling attention to and routing visual input, this region may be computing higher order information present in stimuli and then signaling to other regions, such as the frontal association areas when greater resources are required for further processing, as in the case of subtle gestures and those with communicative intent (Kaiser and Shiffrar, 2009; Thurman et al., 2016). Previous research has shown that this region is more engaged by gestures that were rated by participants as more communicative (Redcay et al., 2016). In our sample, this system appears to function in this manner for both groups in left pSTS, while the ASD group exhibited a different pattern in right pSTS. Atypical patterns of brain lateralization are often observed in ASD and appear most commonly in temporal language-specific circuits (Knaus et al., 2010; Nielsen et al., 2014; Wei et al., 2018). While the gestures presented in the task are not explicit language, they do represent a form of communication and the observed atypical lateralization may relate to similar underlying mechanisms of dysfunction. Research has demonstrated both right- and left-ward dominance of these differential hemispheric effects, but our results align with previous work showing decreased functional connectivity during BM perception specific to the right pSTS in ASD (Jack et al., 2017). Given that social perception tends to be right-lateralized in this region, it is unsurprising that this is where our group differences are observed.

While direct comparisons between groups revealed no regions of difference above threshold, the TD group did show additional activity in some regions such as supplementary motor area and premotor cortex, areas involved in the planning of motor actions. These results provide additional support for the notion that early intact processing in LOTC and pSTS regions allow further processing to activate motor representations that may enhance encoding of gestures and improve subsequent performance.

### Gesture Recognition

The fMRI task revealed no group differences in reaction time or accuracy, suggesting that groups were matched on their ability and the speed at which they recognized gestures. Contrary to our predictions, those with ASD did not require more exaggerated movement to recognize gestures. However, previous literature examining BM perception using point-light displays has shown a similar absence of behavioral group differences in spite of differential neural activity (Freitag et al., 2008; Kröger et al., 2014). Furthermore, deficits in recognition tend to occur for more complex stimuli or that involving emotional content (Alaerts et al., 2014; Krüger et al., 2018). The simplicity of the stimuli and the two-item forced-choice task was perhaps not complex or challenging enough to detect differences in gesture recognition abilities.

There was, however, differential behavioral sensitivity based on gesture type and movement intensity across both groups. With respect to gesture type, participants were less accurate and took longer to identify communicative gestures compared to functional ones, irrespective of diagnosis. It is possible that communicative gestures were more ambiguous to recognize; for example, gestures such as “wave,” “scold,” and “come here” all involve repetitive movement of the right arm around eye-level. By comparison, functional gestures, where the movement was distinct (e.g., driving, knocking), were more easily distinguished, which could explain both increased reaction times and lower accuracy. These behavioral findings are also consistent with the increased neural activity for communicative compared to functional gestures, suggesting that greater neural and cognitive resources were required to process gestures that were social in nature.

The behavioral measures presented a more complicated picture regarding the effect of movement intensity. The subtle gestures took the longest time to recognize compared to both mid-level and exaggerated gestures, which may indicate that subtlety of movement inhibited the timing of the response while greater overtness triggered a rapid response. With respect to accuracy, however, participants were most accurate at recognizing the mid-level gestures compared to both subtle and exaggerated gestures. This may be attributable to the fact that these mid-level gestures were the most natural in the scale of their representation (i.e., did not contain motion that was muted or exaggerative), thus more familiar to and more accurately recognized by participants.

### Gesture Production

In our examination of gesture production abilities, we found that participants with ASD demonstrated poorer overall performance, as hypothesized. This work is consistent with previous research examining deficits in gesturing elicited through command, in which the ASD group showed greater orientation errors (Dewey et al., 2007). This study used a similar paradigm including some of the same action gestures (e.g., brush teeth, cut paper with scissors), so the parallel results are unsurprising.

Individuals with ASD demonstrated specific impairment in hand posture during this task. Qualitatively, this deficit was evident as abnormal flipping of the wrists or unusual finger grasping patterns. Autism-specific hand stereotypies can often be distinguished by their involvement with objects (Goldman and Temudo, 2012) and though no objects were used in our task, actions involving mimed use of objects is often where oddities were observed. This finding supports existing research showing impairments in imitation of hand and finger gestures (Freitag et al., 2006; Biscaldi et al., 2014). Those studies, however, used meaningless gestures without social or tool-related context, so the current study extends these findings of hand gestural abnormalities to common and familiar actions. Interestingly, one of the most commonly noted “orientation errors” in Dewey et al. (2007) was an incorrect rotation of the palm of the hand relative to the arm or to the appropriate plane of movement. The observed abnormalities in previous research and during performance on our task may also relate to repetitive hand/finger mannerisms and stereotypies often observed in ASD, such as hand flapping and twisting wrists.

No differences were observed for other codes such as limb movement, body position and tempo. One possibility is that representation of the more overt, observable aspects of gestures, as measured by these codes, are encoded and performed equally well by both groups, while subtler aspects of hand and finger posture may be more challenging for those with ASD. As previously noted, the BM deficit in ASD is one of degree, such that those with ASD require greater signal to detect BM. The nature of this perceptual deficit may manifest as faulty encoding and representation of the subtle aspects of action movements, resulting in a specific deficit in performance of these components.

Individuals with ASD showed greater use of environmental context, including walls, chairs, and a blackboard while performing their gestures. This suggests that those with ASD may have had a greater reliance on these items as a tool for communication, instead of representing the gesture with their body alone. However, we found no correlation between use of environmental context and any individual code or total score, suggesting that the environment was not utilized successfully to improve performance.

Our task showed no group differences in the “gaze” code, which required participants to make at least one clear social reference to the confederate observer while performing the action. Given the amount of research demonstrating reduced eye contact and joint attention in ASD (e.g., Bhat et al., 2010), this finding was unexpected. Our data suggest that participants with ASD performed at least basic social referencing skills to the same degree as their TD counterparts. However, our coding scheme did not assess quality, such as duration, or quantity of social referencing behavior, and thus may have failed to detect a real effect in this domain.

### Relationships Between Measures

The examination of the relationship between measures of gesture processing and production suggested that in neurotypical individuals, heightened neural processing of gestures is associated with better gesture production. This association was significant within the LOTC and AON regions, however the latter appears to be accounted for by age. This finding suggests that these areas may be part of a neural mechanism linking perceptual representations of gestures to performance of these actions. There is considerable research establishing the AON in this role, functionally supporting the encoding and representation of both action observation and execution (Oberman and Ramachandran, 2007; Schippers et al., 2009; Van Overwalle and Baetens, 2009; Wadsworth et al., 2017), while LOTC’s role in this capacity is just emerging (Zimmermann et al., 2018) and may warrant further investigation. No association between activity in bilateral pSTS and gesture performance was detected, which may suggest that this region is less relevant for gesture production compared to the other two areas. Previous research supports its role in controlling attention to and routing socially relevant visual stimuli, making it an ideal candidate for activation in our gesture task, however, its role in the production of action has been yet been fully elucidated. The absence of any correlation in the ASD group may reflect some dysfunction in the perception-action coupling system, however, the small sample size may also contribute to the lack of statistically significant findings.

There were also relationships between gesture performance and both measures of social functioning in the TD group, with similar trends in the ASD group. These results indicate that compromised gesturing abilities are related to greater social impairment in both groups. These findings strengthen an extensive body of literature linking gesture and imitation deficits to severity of social impairment (Dziuk et al., 2007; Nebel et al., 2016), both core features of ASD. Individuals who are better able to communicate using gestures, as required by the task, demonstrate greater social and communicative skills.

Social impairment was also inversely related to neural activity during gesture processing, suggesting that increased processing of gestures may be related to better social outcomes. This relationship was significant for left LOTC and AON activity in the TD group, supporting previous research linking attenuated neural activity during BM processing to greater severity of social symptoms (Kaiser et al., 2010). Past work has also shown these associations in ASD, and authors have suggested that a faulty perceptual system could lead to inaccurate representations of the actions of others resulting in social impairment (Oberman et al., 2005; Dapretto et al., 2006; Iacoboni and Dapretto, 2006; Oberman and Ramachandran, 2007). Given the lack of statistically significant difference between the correlations in ASD and TD groups, it is likely that the null finding in the ASD group is attributable to the small sample size.

Impaired motor coordination was associated with poorer overall performance of gestures, significant only in the TD group. Given that higher DCDQ scores reflect greater coordination of movement on activities like throwing a ball, writing or cutting paper, an association with performance of related gestures was expected. Previous studies have shown relationships between motor abilities and gestural skills in TD groups, consistent with our findings, but also in ASD (Dewey et al., 2007; Zachor et al., 2010; Biscaldi et al., 2014), suggesting that our sample size may have lacked power. There were no associations between motor functioning and neural activity in any ROI region, which is unsurprising given that these regions, with the exception of a few areas in the AON mask (i.e., IFG), are not associated with motor planning and execution.

There are a few limitations to note. Given that not all participants completed the fMRI task and the need to exclude poor data, our sample size in the whole brain and ROI analyses was relatively small. Additionally, the absence of a localizer task precluded our analyses from pinpointing with anatomical specificity the exact functional region within the LOTC where whole brain activation clusters were found. Future research could employ distinct BM and body form localizers to dissociate EBA from MT+, as other studies have done (Ross, 2014). Another limitation of the study was that it employed a novel gesture task, as there was not a validated task suitable for evaluating performance of complex action gestures in the manner we wanted. Furthermore, the gestures observed in the fMRI task were different from the gestures performed during the charades task and thus we lack a one-to-one comparison of actions through both execution and observation. Instead, the conclusions drawn about associations between gesture processing and production represent a more general trend indicating action-observation coupling.

## Conclusion

This study provides some evidence for a pathway linking impairment in BM perception and gestural deficits. The findings extend our understanding of ASD and have important translational potential, offering insight into how an impaired BM detection system may manifest in the broader social deficits observed in ASD. Knowledge about the altered neural and cognitive mechanisms involved in processing non-verbal social cues may help guide interventions focused on increasing children’s attention to social input. For example, one recent study demonstrated that fMRI-based stratification of activity in response to BM in neural circuits underlying social information processing (STS, inferior parietal cortex) accurately predicted response to evidence-based behavioral treatment, namely, Pivotal Response Training. The authors underscore the potential of this measure to be used as a sensitive, objective neurobiological marker to identify subgroups of young children likely to respond to specific treatments within a sensitive window of opportunity (Yang et al., 2016).

The present study demonstrated an interesting pattern of action perception coupling but further research will be necessary to disentangle the nature of the relationships between gesture processing, production and social skills. Likely, there is a dynamic and bidirectional interplay between the various components, with perceptual deficits leading to cascading effects on atypical social development, in conjunction with social attention and preference influencing patterns of neural activity during observation of social stimuli. Future research should recruit larger samples and employ additional methodologies and paradigms, that may be more suitable for children who are younger and lower functioning in order to augment our understanding of the complex pathways linking BM processing, gestural abilities and social responsiveness.

## Data Availability Statement

The datasets generated for this study are available on request to the corresponding author.

## Ethics Statement

The studies involving human participants were reviewed and approved by University of California, Davis Institutional Review Board. Written informed consent to participate in this study was provided by the participants’ legal guardian.

## Author Contributions

SR and JP conceived and designed the study. MN created the stimuli. JP recruited the participants, collected the data, and performed the initial analyses. EF and EP created the coding protocol and coded all videos. EP performed the behavioral analyses. EF performed the fMRI and correlation analyses, interpreted the results, and drafted the manuscript. All authors read and reviewed the final manuscript.

## Conflict of Interest

The authors declare that the research was conducted in the absence of any commercial or financial relationships that could be construed as a potential conflict of interest.

## Footnotes

1 https://www.fil.ion.ucl.ac.uk/spm/software/spm12

2 http://marsbar.sourceforge.net
