# Supplementary material for: Neural Processing and Production of Gesture in Children and Adolescents With Autism Spectrum Disorder
Source: Front Psychol. 2020 Jan 22;10:3045. doi: 10.3389/fpsyg.2019.03045 (PMC6987472; doi:10.3389/fpsyg.2019.03045)
Supplement: Supplementary file 1 [file Table_1.DOCX]

**Appendix**

**Gesture coding scheme**

1. **Events acted out**

**2** All events completed

**1** One event missing

**0** More than one/all events missing

1. **Gaze**

**2** Fully engaged with partner, shows at least one clear example of a social reference during performance of the gesture.

**1** Some engagement with partner although this is reduced compared to what would typically be expected. Perhaps only references partner after gesture is completed.

**0** Does not show any social referencing to partner.

1. **Body positioning**

**2** Uses a body posture that is both appropriate for the gesture and suitable for the partner’s position in the room. Do not code use of limbs here, merely the positioning of the whole body.

**1** Uses a somewhat abnormal posture either for the gesture being performed or for the position of the partner, e.g. angles body somewhat away from partner or center of gesture.

**0** Faces away from partner or performs gesture outside partner’s field of view.

1. **Limb movement** (see specific requirements for each gesture)

**2** All components correct.

**1** One component is incorrect/ missing but the rest are correct.

**0** Multiple components are incorrect/missing.

1. **Hand posture**

**2** Able to accurately perform all hand postures required for the movements conveyed. No abnormal or unusual quality to any.

**1** Shows some elements of abnormality or error but can also demonstrate correct hand posture at times. Alternatively, a participant who only displays a minor abnormality throughout should also be coded here.

**0** Hand posture is abnormal or erroneous throughout gesture or features a major error or abnormality.

1. **Use of space**

**2** Used the space around them appropriately. Gesture is not overly large, too small or distorted around the body.

**1** Some abnormality in the use of space. Gesture may be a little on the small side, or overly overt. Limbs may be contorted in a slightly unusual manner.

**0** Major abnormality in the use of space. Gesture may be extremely small or large. In the latter, hands may reach outer limits of reaching space. Additionally, gestures that involve highly abnormal contortion of the limbs should be coded here.

1. **Tempo**

**2** Gesture is performed at a pace that is realistic and appropriate to the movements conveyed

**1** Semi-realistic and/or appropriate pace. A gesture that demonstrates a change in tempo during its depiction that is not functional should also be coded here.

**0** Not performed at a pace that is realistic or appropriate to the movements conveyed. Gestures that contain components that are jerky or unintegrated into the whole movement should be coded here.

1. **Meaning**

**2** Gesture clearly resembles its meaning.

**1** You have a reasonable idea of the gesture’s meaning. Perhaps you might not have been 100% sure had you not known beforehand, but you would have a reasonable idea.

**0** The gesture does not clearly resemble its meaning, and it could be as equally conceivable that they are performing a different action.

1. **Overall quality**

**2** Gesture is well-performed and precise.

**1** Gesture has some errors and/or abnormal qualities, although is generally accurate.

**0** Gesture is poor and has major errors and/or abnormalities in how it is executed.

**Additional codes**

1. Uses own body as object, e.g. hand as paper? **Y N**
2. Mimes using an imaginary object, e.g. holding a toothbrush? **Y N**
3. Uses environmental context, e.g. uses lab furniture or wall? **Y N**
4. Embellishes gesture with additional context, e.g. licks ice cream? **Y N**
5. Makes multiple attempts to perform gesture? **Y N**

**Consensus on scoring of gestures**

A Disagreement Score between the scorers was determined by subtraction of scores across all items labelled 1-8 above. Any trials where the Disagreement Score was greater than three were discussed by the two scorers until consensus was reached (19% of all trials) and averaging was used to integrate the scores on all other trials. Item 9 above, which required the overall quality of the gesture to be scored, was not entered into the Disagreement Score as this code was designed to reflect the subjective assessment of the scorer and was thus unlikely to have a high degree of convergence, and so these scores were simply averaged across the two scorers. Any trials where the scorers disagreed on items labelled 10-14 were discussed until consensus was reached.
